# Risk factors for Lyme disease stage and manifestation using electronic health records

**DOI:** 10.1186/s12879-021-06959-y

**Published:** 2021-12-20

**Authors:** Katherine A. Moon, Jonathan S. Pollak, Melissa N. Poulsen, Christopher D. Heaney, Annemarie G. Hirsch, Brian S. Schwartz

**Affiliations:** 1 Department of Environmental Health and Engineering, Johns Hopkins Bloomberg School of Public Health, 615 N Wolfe St., W7604, Baltimore, MD USA; 2 Department of Population Health Sciences, Geisinger, Danville, PA USA; 3 Department of Epidemiology, Johns Hopkins Bloomberg School of Public Health, Baltimore, MD USA; 4 Department of Medicine, Johns Hopkins School of Medicine, Baltimore, MD USA

**Keywords:** Lyme disease, Tick-borne disease, Electronic health records, Epidemiology, Disease stage, Disseminated Lyme disease

## Abstract

**Background:**

Little is known about risk factors for early (e.g., erythema migrans) and disseminated Lyme disease manifestations, such as arthritis, neurological complications, and carditis. No study has used both diagnoses and free text to classify Lyme disease by disease stage and manifestation.

**Methods:**

We identified Lyme disease cases in 2012–2016 in the electronic health record (EHR) of a large, integrated health system in Pennsylvania. We developed a rule-based text-matching algorithm using regular expressions to extract clinical data from free text. Lyme disease cases were then classified by stage and manifestation using data from both diagnoses and free text. Among cases classified by stage, we evaluated individual, community, and health care variables as predictors of disseminated stage (vs. early) disease using Poisson regression models with robust errors. Final models adjusted for sociodemographic factors, receipt of Medical Assistance (i.e., Medicaid, a proxy for low socioeconomic status), primary care contact, setting of diagnosis, season of diagnosis, and urban/rural status.

**Results:**

Among 7310 cases of Lyme disease, we classified 62% by stage. Overall, 23% were classified using both diagnoses and text, 26% were classified using diagnoses only, and 13% were classified using text only. Among the staged diagnoses (n = 4530), 30% were disseminated stage (762 arthritis, 426 neurological manifestations, 76 carditis, 95 secondary erythema migrans, and 76 other manifestations). In adjusted models, we found that persons on Medical Assistance at least 50% of time under observation, compared to never users, had a higher risk (risk ratio [95% confidence interval]) of disseminated Lyme disease (1.20 [1.05, 1.37]). Primary care contact (0.59 [0.54, 0.64]) and diagnosis in the urgent care (0.22 [0.17, 0.29]), compared to the outpatient setting, were associated with lower risk of disseminated Lyme disease.

**Conclusions:**

The associations between insurance payor, primary care status, and diagnostic setting with disseminated Lyme disease suggest that lower socioeconomic status and less health care access could be linked with disseminated stage Lyme disease. Intervening on these factors could reduce the individual and health care burden of disseminated Lyme disease. Our findings demonstrate the value of both diagnostic and narrative text data to identify Lyme disease manifestations in the EHR.

**Supplementary Information:**

The online version contains supplementary material available at 10.1186/s12879-021-06959-y.

## Introduction

Lyme disease, caused by the bacteria *Borrelia burgdorferi*, is transmitted to humans through an infected tick bite. Most Lyme disease cases occur in the northeastern and midwestern United States (US); however, the geographic distribution of tick vectors and incidence of tick-borne diseases has been expanding [1]. Recent estimates suggest that Lyme disease was treated and diagnosed in approximately 476,000 persons annually between 2010 and 2018 [2]. Lyme disease is commonly categorized into two main stages: early stage, where infection is localized (e.g., an expanding erythema migrans lesion), or disseminated stage, where infection has spread beyond the initial bite location [3–5]. Untreated, Lyme disease typically progresses from a localized skin infection, often with systemic non-specific symptoms (e.g., fatigue, headache), to various disseminated infections. Most uncomplicated cases fully recover after antibiotic treatment [6]. Disseminated manifestations range from secondary erythema migrans rashes, acute neurological effects (e.g., facial palsy, meningitis, radiculopathy), and carditis, which usually occur weeks to months after infection, to Lyme arthritis, the most common late disseminated infection, which usually occurs months to years after infection [3]. Only 30–50% of cases recall being bitten by a tick [7], and serological tests have low sensitivity early in the disease course [6]; therefore, early diagnosis largely relies on recognition of a characteristic erythema migrans rash. However, up to 20–30% of cases may not have erythema migrans [7], and atypical presentations of erythema migrans are more likely to be misdiagnosed [8, 9].

It is not well understood why some patients do not develop erythema migrans, or why others develop specific disseminated manifestations. Few epidemiologic studies have examined risk factors for disseminated stage Lyme disease, compared to early disease, or risk factors for developing specific manifestations. These studies have largely evaluated relations with demographic variables (age, sex, race, ethnicity), season of diagnosis, or with specific presenting clinical signs and symptoms [10–14]. Most studies identified Lyme disease cases and manifestations from surveillance data [11, 13, 15, 16] or from hospital or clinic data [14, 17, 18]. A recent study used billing codes in insurance claims from high-incidence states to identify disseminated Lyme disease manifestations, including arthritis, facial palsy, carditis, complete heart block, and meningitis, and evaluated trends in age, sex, seasonality, and hospitalization [10].

Like claims data, electronic health record (EHR) data contain rich longitudinal clinical data on diagnoses, medications, and laboratory test orders; however, EHR data also contain the results of laboratory testing and narrative free text notes added by a health care provider. Most EHR studies identify clinical outcomes using structured diagnosis data, such as diagnosis or billing codes; however, natural language processing algorithms are increasingly being developed to extract valuable clinical data from narrative text notes [19]. EHR and claims-based studies of Lyme disease have largely used diagnosis data to identify Lyme disease [2, 20–24]. Although prior EHR studies have identified erythema migrans using keyword-based text matching algorithms [25, 26], no studies have used both diagnosis data and narrative free text data to comprehensively classify Lyme disease diagnoses by stage of disease and manifestation. Classifying Lyme disease in the EHR will allow for future research to evaluate the effectiveness of intervening on modifiable risk factors for disseminated stage diagnosis that can reduce the burden of Lyme disease.

This study had two aims. First, we evaluated the classification ability of diagnosis data and free text from encounter notes to classify Lyme disease by stage of disease and clinical manifestation. Second, we examined whether individual, community-level, and health care factors were associated with disseminated stage compared to early stage Lyme disease.

## Methods

### Study population

Geisinger is an integrated health system that provides primary, specialty, urgent, and emergency health care services at community practice clinics and hospitals in central and northeastern Pennsylvania. For this study, we started with 1,128,671 patients in the Geisinger EHR (primary care and non-primary care) between January 2012 through December 2016 and whose most recent address was within Geisinger’s primary service area and surrounding counties (38 counties).

### Lyme disease case identification

We identified persons diagnosed with Lyme disease by the presence of at least one diagnosis for Lyme disease, using both Epic (Verona, WI) electronic diagnosis group (EDG) names or International Classification of Diseases Ninth and Tenth Revision, Clinical Modification (ICD-9-CM 088.81 and ICD-10-CM A69.2x) codes (Additional file 1: Table S1). Relevant EDG names were identified using an iterative keyword search reviewed by a clinician (BSS). To focus on new diagnoses, we excluded persons with diagnoses indicating a history of Lyme disease up until six months prior to the index diagnosis. The Geisinger Institutional Review Board approved this study and waived informed consent. Hereafter, we will use the term “case” to describe persons who were diagnosed with Lyme disease.

### Classification of Lyme disease diagnoses by clinical stage and manifestation

**Fig. 1:**
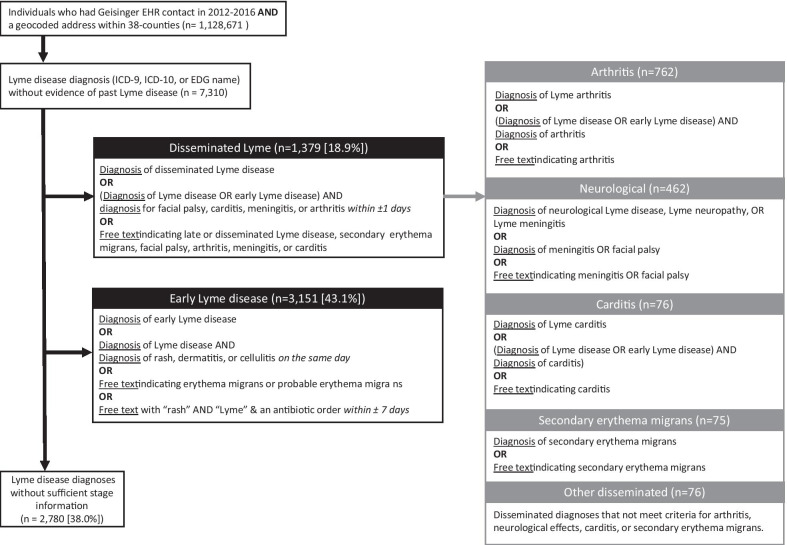
Algorithm to classify Lyme disease cases by clinical stage and manifestation. *ICD* International Classification of Disease, *EDG* electronic diagnosis group

We categorized Lyme disease cases by clinical stage and manifestation using two sources, diagnoses associated with either encounters or medication orders, and narrative free text associated with an in-person outpatient, emergency, or urgent care encounter (Fig. 1). We classified Lyme disease cases as early localized (erythema migrans) or disseminated stage disease. Among those with disseminated stage disease, we further classified cases into four non-mutually exclusive groups: arthritis, neurological manifestations, carditis, and “other disseminated” manifestations. Manifestations were assigned when recorded within a day or less from a generic Lyme disease code.

We examined diagnoses (EDG names, ICD-10, ICD-9) for Lyme disease (Additional file 1: Table S1) and related co-diagnoses (Additional file 1: Table S2) from inpatient, outpatient, emergency, or urgent care encounters and from medication orders, and narrative free text from outpatient, emergency, or urgent care encounters within 31 days before or after the index Lyme disease diagnosis. EDG names allow for a higher level of diagnostic detail. EDG names can identify all Lyme disease manifestations, including erythema migrans, which cannot be identified using ICD codes. Based on the information related to stage and manifestation, diagnoses could be categorized as either: (1) a recognized manifestation of Lyme disease (e.g., Lyme arthritis), (2) information on stage but not manifestation (e.g., “early localized Lyme infection”); or (3) no information on disease stage or manifestation (e.g., “Lyme disease”). In cases with only generic Lyme disease diagnoses, we identified diagnoses related to manifestations of Lyme disease (e.g., rash, arthritis, meningitis) in order to determine stage or manifestation.

To extract diagnosis information from free text, we used regular expressions in Stata (MP, Version 15.1) to match words and phrases indicating Lyme disease stage or manifestation. Key words and phrases were developed using an iterative process led by a clinician (BSS), including a review of a subset of notes from a variety of years and settings to account for common misspellings and abbreviations. We identified strings of text before and after matched keywords to exclude where the concept was negated (e.g., “no sign of”), temporally unrelated, (e.g., “history of”), or unrelated to the subject (e.g., “husband has arthritis”). In cases with evidence for both early and disseminated stage Lyme disease, cases were classified as disseminated stage under the assumption that the presenting signs and symptoms were the later disseminated manifestations.

### Validation of EHR algorithm for early and disseminated stage Lyme disease

We conducted a validation study to calculate the positive predictive value (PPV) of our EHR algorithm to classify Lyme disease cases as either early stage or disseminated stage. The PPV was calculated as the percentage of persons where the algorithm-assigned stage of Lyme disease was the same as the manual review-assigned stage of Lyme disease (reference standard). Two investigators (B.S and K.M) manually reviewed the EHR records. EHR documentation reviewed included demographics, health insurance payor, diagnoses (e.g., ICD codes and EDG names), Lyme disease serologic testing results, antibiotic medication orders, problem list, and free text clinical notes. Among Lyme disease cases that the EHR algorithm classified by stage of Lyme disease, we randomly selected a subset of 100 persons, 50 persons assigned as early stage and 50 persons assigned as disseminated stage. We stratified selection by year of diagnosis (10 persons per each of the 5 study years) because patterns of documentation, including for Lyme disease, have changed in the Geisinger EHR over time. We calculated 95% confidence intervals for the PPV using a binomial test.

### Other variables

We extracted individual-level covariates from the EHR, including age, sex, race, ethnicity, setting of diagnosis, and season of diagnosis. Exposure to disease-carrying ticks is most likely in the late spring and summer [7, 27–29], and we hypothesized that compared to early stage cases, disseminated stage diagnosis would be more likely during other seasons. Individuals were defined as having primary care contact if they had two or more outpatient primary care encounters (e.g., family practice, internal medicine, pediatrics, and obstetrics/gynecology departments) prior to Lyme disease diagnosis. In Pennsylvania, Medical Assistance (i.e., Medicaid and Children’s Health Insurance Program [CHIP]), pays for health care services for eligible individuals [30]. We considered the percentage of time an individual used Medical Assistance prior to Lyme disease diagnosis as a surrogate for household socioeconomic status (SES) [31].

In early stage Lyme disease, serology is not recommended due to a high probability of false negatives [32]. Blood samples drawn more than four weeks after disease onset are recommended to be tested for IgG, not IgM, because of high risk of false positive results with IgM at this stage [33, 34]. Consistent with prior analyses of the Geisinger EHR [21], we defined a positive Lyme disease serological test as either an IgG positive test (either alone, with a positive enzyme immunoassay [EIA], or with negative EIA), or an IgM positive test with a positive EIA within 30 days of diagnosis. The vast majority of Lyme disease diagnoses with positive IgG Western blots had a positive EIA (96.4%), meeting the recommendations of the CDC diagnostic criteria ((CDC), 1995). For the cases with positive IgG Western blots without EIA (3.5%) or with a negative EIA (0.1%), we thought it was possible that the initial positive EIA was obtained and recorded outside of Geisinger and thus categorized these test results as seropositive. We defined evidence of appropriate treatment as a medication order for an appropriate antibiotic [5, 24] within 30 days before or after the Lyme disease diagnosis date.

We used geocoded addresses to assign community variables. Most addresses (88%) were geocoded to the street address; otherwise, we assigned the zip code centroid. We used the U.S. Census Bureau categorization of urbanized area (50,000 or more people), urban clusters (at least 2500 and less than 50,000 people), or rural areas (persons not in urbanized areas or urban clusters) [35]. We hypothesized that disseminated Lyme disease may be more likely in rural areas, due to higher systemic and individual barriers to health care in rural areas compared to urban areas [36].

### Statistical analysis

We first used descriptive analyses to evaluate selected individual, community, and health care variables among Lyme disease cases classified by stage and by manifestation. We evaluated how these variables differed across classified and unclassified cases, and by data source (e.g., both diagnoses and free text, diagnoses only, or free text only). To identify risk factors for disseminated disease, we conducted a case-only analysis of disseminated stage compared to early stage Lyme disease cases. With multivariable Poisson regression models using generalized estimating equations (GEE) with robust standard errors, we estimated risk ratios (RR) [37] of factors associated with disseminated stage Lyme disease (vs. early stage) and with specific disseminated manifestations (arthritis, neurological manifestations, carditis, secondary erythema migrans, or unspecified “other disseminated” manifestations vs. early stage). All models specified robust standard errors clustered within community (township, borough, or city census tract). Initial models were adjusted for a priori potential confounding variables. Final models included age (< 10, 10 to < 20, 20 to < 30, 30 to < 50, 50 to < 70 [reference], and 70+ years), sex (female, male [reference]), race (non-white, white [reference]), use of Medical Assistance (0% [reference], > 0 to < 50%, and 50–100% of prior observation time), primary care contact (yes, no [reference]), setting of diagnosis (outpatient [reference], urgent care, emergency, inpatient), season of diagnosis (winter, spring, summer [reference], fall), and urban/rural status (urban, urban cluster, or rural area [reference]). In exploratory analyses, we evaluated whether sex, Medical Assistance, or season modified relations between setting of diagnosis and Lyme disease stage by including relevant cross-product terms in separate models, fully adjusted for all covariates. We conducted statistical analyses in Stata (MP, Version 14).

To address potential outcome misclassification, we evaluated a model in which disseminated cases were required to have positive serology (IgG or IgM). We hypothesized that requiring a positive serology result would exclude false positive cases (e.g., due to coding errors or “rule-out” laboratory tests) but would also exclude some true cases (e.g., in which serology was conducted outside Geisinger or tested before antibodies were detectable).

## Results

### Lyme disease cases classified by stage and manifestation

**Fig. 2:**
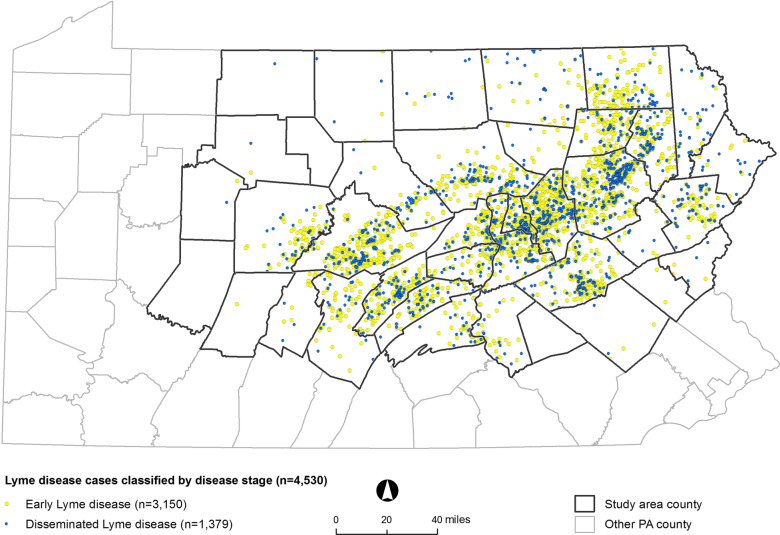
Spatial distribution of Lyme disease cases classified as early and disseminated stages of Lyme disease

We identified 7310 cases of Lyme disease between 2012 and 2016 that met inclusion criteria (Fig. 1). Using diagnoses and narrative free text, we classified 4530 cases (62%) as early or disseminated stage. Of the classified cases, 70% were classified as early stage disease. Of the 1,359 disseminated cases, Lyme arthritis was the most common manifestation (55%), followed by neurological manifestations (34%), carditis (6%), and secondary erythema migrans (7%). Diagnoses classified as disseminated stage that did not meet criteria for arthritis, neurological effects, carditis, or secondary erythema migrans were classified as “other disseminated” manifestations (6%). In a validation sample of 50 early and 50 disseminated cases, we found that the PPV of early stage disease was 92% (95% CI 81–98%) and the PPV of disseminated stage Lyme disease was 88% (95% CI 76–95%). Lyme disease cases were distributed across the study area in central and northeastern Pennsylvania (Fig. 2).

### Classified Lyme disease cases by source of staging information

Among all persons diagnosed with Lyme disease (n = 7310), 23% were classified using information in both diagnoses and text, 26% were classified using information in diagnoses only, 13% were classified using information in text only, and 38% were not classified. Overall, diagnoses were able to classify 49%, compared to 36% with free text. The percentage of diagnoses classified increased over the study period: persons classified by stage using diagnoses increased from 43 to 50% and cases classified by text increased from 21 to 55%.

Among Lyme disease cases classified by stage (n = 4530), we observed notable patterns of sources of staging data over time and by participant characteristics (Additional file 1: Table S3). Over the study period, the percentage of classified cases with information in diagnoses alone decreased from 60 to 21%, while cases classified by text alone increased from 19 to 27%. Cases with staging-relevant data from both diagnoses and text were younger, while cases with staging data only in text were older. The most common source of data for staging varied by setting of diagnosis, primary care contact, and time using Medical Assistance.

### Cases classified by stage compared to cases not classified by stage

Lyme disease cases who were classified by stage (62%, n = 4530) differed from those who were not (38%) (Additional file 1: Table S4). Over the study period, the percentage of cases classified increased from 54 to 69%. Classified cases were younger, more likely to be diagnosed in the summer, and more likely to live in a rural area. Cases with sufficient information relevant to staging were more likely to be diagnosed in urgent care, less likely to be diagnosed in the emergency department, and more likely to have primary care contact.

### Characteristics of early and disseminated stage Lyme disease cases

We observed several differences in individual, community, and healthcare characteristics across Lyme disease stages and manifestations (Table 1). The secondary erythema migrans group and the “other disseminated” manifestations group had the lowest median age, followed by arthritis, and neurological manifestations and carditis. Lyme carditis was less commonly observed in females, whereas other manifestations were more equally distributed among men and women. Lyme disease cases were most commonly diagnosed in outpatient settings; however, Lyme carditis was more evenly split between inpatient and outpatient settings. Diagnosis in summer was most common across manifestations, except for Lyme arthritis, which was diagnosed relatively equally in fall and summer. For most early (94.5%) and disseminated (82.3%) cases, an appropriate antibiotic was ordered within 30 days. Among disseminated stage cases, when serology is most likely recommended, most cases (59%) had at least one serologic test order within 30 days. Just under half of all disseminated cases had positive IgG or IgM serology (45% overall, 77% of cases with test orders).

**Table 1:** Selected characteristics of 3151 early stage and 1379 disseminated stage Lyme disease cases in the Geisinger electronic health record, 2012–2016

Characteristic	Early Lyme disease	Disseminated Lyme disease	Disseminated manifestations				
			Arthritis	Neurological manifestations	Carditis	Secondary erythema migrans	Other disseminated manifestations
	n = 3151	n = 1379	n = 762	n = 426	n = 76	n = 95	n = 76
Sex, female, n (%)	1426 (45.3%)	618 (44.8%)	346 (45.4%)	194 (45.5%)	21 (27.6%)	47 (49.5%)	40 (52.6%)
Age, years, median (IQR)	40.7 (12.9, 59.8)	31.5 (10.8, 57.3)	28.6 (9.8, 57.2)	37.8 (14.9, 57.2)	39.1 (20.7, 58.9)	17.9 (7.5, 57.4)	17.5 (8.7, 53.4)
Age, n (%)							
< 10 years	627 (19.9%)	311 (22.6%)	196 (25.7%)	66 (15.5%)	3 (4.0%)	32 (33.7%)	25 (32.9%)
10 to < 20 years	384 (12.2%)	261 (18.9%)	141 (18.5%)	77 (18.1%)	15 (19.7%)	18 (18.9%)	16 (21.1%)
20 to < 30 years	244 (7.7%)	100 (7.3%)	51 (6.7%)	35 (8.2%)	13 (17.1%)	7 (7.4%)	1 (1.3%)
30 to < 50 years	642 (20.4%)	249 (18.1%)	118 (15.5%)	101 (23.7%)	18 (23.7%)	8 (8.4%)	13 (17.1%)
50 to < 70 years	952 (30.2%)	333 (24.2%)	188 (24.7%)	110 (25.8%)	19 (25.0%)	20 (21.1%)	15 (19.7%)
> 70 years	302 (9.6%)	125 (9.1%)	68 (8.9%)	37 (8.7%)	8 (10.5%)	10 (10.5%)	6 (7.9%)
Race/ethnicity, non-White, n (%)^a^	34 (1.1%)	25 (1.8%)	14 (1.8%)	8 (1.9%)	0 (0%)	1 (1.1%)	3 (3.9%)
Year of diagnosis, n (%)							
2012	322 (10.2%)	234 (17.0%)	163 (21.4%)	51 (12.0%)	6 (7.9%)	12 (12.6%)	9 (11.8%)
2013	527 (16.7%)	235 (17.0%)	134 (17.6%)	78 (18.3%)	17 (22.4%)	8 (8.4%)	9 (11.8%)
2014	607 (19.3%)	278 (20.2%)	149 (19.6%)	89 (20.1%)	19 (25%)	24 (25.3%)	10 (13.2%)
2015	765 (24.3%)	278 (20.2%)	145 (19.0%)	96 (22.5%)	11 (14.5%)	20 (21.1%)	15 (19.7%)
2016	90 (29.5%)	354 (25.7%)	171 (22.4%)	112 (26.3%)	23 (30.3%)	31 (32.6%)	33 (43.4%)
Season of diagnosis, n (%)							
Winter (Dec–Feb)	109 (3.5%)	181 (13.1%)	145 (19.0%)	30 (7.0%)	2 (2.6%)	4 (4.2%)	5 (6.6%)
Spring (March–May)	356 (11.3%)	181 (13.1%)	140 (18.4%)	36 (8.5%)	4 (5.2%)	8 (8.4%)	3 (3.9%)
Summer (June–Aug)	2110 (67.0%)	653 (47.4%)	222 (29.1%)	289 (67.8%)	54 (71.1%)	69 (72.6%)	52 (68.4%)
Fall (Sept–Nov)	576 (18.3%)	364 (26.4%)	255 (33.5%)	71 (16.7%)	16 (21.1%)	14 (14.7%)	16 (21.1%)
Setting of diagnosis, n (%)							
Outpatient	2312 (73.4%)	1110 (80.5%)	667 (87.5%)	310 (72.8%)	33 (43.4%)	80 (84.2%)	66 (86.8%)
Urgent care	667 (21.5%)	56 (4.1%)	32 (4.2%)	13 (3.1%)	0 (0%)	11 (11.6%)	1 (1.3%)
Emergency	131 (4.2%)	77 (5.6%)	28 (3.7%)	39 (9.2%)	4 (5.3%)	4 (4.2%)	3 (3.9%)
Inpatient	31 (1.0%)	136 (9.9%)	35 (4.6%)	64 (15.0%)	39 (51.3%)	0 (0.0%)	6 (7.9%)
Primary care contact, yes, n (%)	2619 (83.1%)	948 (68.8%)	540 (70.9%)	287 (67.4%)	43 (56.6%)	80 (84.2%)	38 (50.0%)
Medical assistance, n (%)							
0% time	2554 (81.1%)	1061 (76.9%)	571 (74.9%)	336 (78.9%)	60 (78.9%)	76 (80.0%)	59 (77.6%)
> 0% to < 50% time	370 (11.7%)	156 (11.3%)	83 (10.9%)	45 (10.6%)	8 (10.5%)	13 (13.7%)	16 (21.1%)
≥ 50% time	227 (7.2%)	162 (11.7%)	108 (14.2%)	45 (10.6%)	8 (10.5%)	6 (6.3%)	1 (1.3%)
Community of residence, n (%)							
Township	2424 (76.9%)	1002 (72.7%)	564 (74.0%)	299 (70.2%)	50 (65.8%)	72 (75.8%)	55 (72.4%)
Borough	613 (19.5%)	309 (22.4%)	163 (21.4%)	97 (22.8%)	24 (31.6%)	22 (23.2%)	19 (25.0%)
City	114 (3.6%)	68 (4.9%)	35 (4.6%)	30 (7.0%)	2 (2.6%)	1 (1.1%)	2 (2.6%)
Urban/rural residence, n (%)							
Rural	1987 (63.1%)	820 (59.5%)	459 (60.2%)	251 (58.9%)	44 (57.9%)	57 (60.0%)	45 (59.2%)
Urban cluster	465 (14.8%)	239 (17.3%)	134 (17.6%)	76 (17.8%)	12 (15.8%)	19 (20.0%)	19 (25.0%)
Urbanized area	699 (22.2%)	320 (23.2%)	169 (22.2%)	99 (23.2%)	20 (26.3%)	19 (20.0%)	12 (15.8%)
Community socioeconomic deprivation, n (%)							
Quartile 1	918 (29.1%)	355 (25.7%)	198 (26.0%)	103 (24.2%)	26 (34.2%)	25 (26.3%)	18 (23.7%)
Quartile 2	1031 (32.7%)	423 (30.7%)	234 (30.7%)	125 (29.3%)	20 (26.3%)	38 (40.0%)	26 (34.2%)
Quartile 3	746 (23.7%)	370 (26.8%)	201 (26.4%)	119 (27.9%)	17 (22.4%)	17 (17.9%)	23 (30.3%)
Quartile 4	456 (14.5%)	231 (16.8%)	129 (16.9%)	79 (18.5%)	13 (17.1%)	15 (15.8%)	9 (11.8%)
Antibiotic order within ± 30 days, n (%)^c^	2977 (94.5%)	1135 (82.3%)	618 (81.1%)	349 (81.9%)	64 (84.2%)	88 (92.6%)	59 (77.6%)
Lyme disease serology order ± 30 days, n (%)	–^b^	810 (58.7%)	468 (61.4%)	240 (56.3%)	36 (47.4%)	42 (44.2%)	34 (44.7%)
IgG+ WB or EIA+/IgM+ WB ± 30 days, n (%)	–^b^	621 (45.0%)	333 (43.7%)	202 (47.4%)	49 (64.5%)	30 (31.6%)	29 (38.2%)

*IQR* interquartile range, *IgG* immunoglobulin G, *IgM* immunoglobulin M, *WB* Western blot, *EIA* enzyme immunoassay

^a^Race was recorded as white, Black or African American, American Indian or Alaska Native, Asian, Native Hawaiian or Pacific Islander, mixed, or unknown. In this sample, non-White race/ethnicity included 42% Black or African American, 31.8% American Indian or Alaska Native, Asian, Native Hawaiian or Pacific Islander, 11.8% mixed race, and 9.1% unknown race

^b^Serological diagnostic testing is not recommended for early stage Lyme disease cases because of a high probability of false negative results before a robust antibody-mediated immune response

^c^Lyme disease-appropriate antibiotic order, as previously described [20]

### Disseminated vs. early stage Lyme disease

In a multivariable model examining risk factors of disseminated versus early stage Lyme disease, we observed several interesting associations (risk ratio [95% confidence interval]) (Table 2). Compared to persons with 0% of time using Medical Assistance, persons who used Medical Assistance for 50% or more time prior to Lyme disease diagnosis had a higher risk of disseminated Lyme disease overall (1.20 [1.05, 1.37]), arthritis (1.37 [1.15, 1.64]), and neurological manifestations (1.22 [0.93, 1.60]), no difference in risk of carditis (1.04 [0.47, 2.27]), but a lower risk of secondary erythema migrans (0.50 [0.24, 1.22]) and other disseminated stage (0.15 [0.02, 1.16]). Individuals with primary care contact had lower risk of other disseminated disease (0.59 [0.54, 0.64]). Compared to outpatient settings, we found that the inpatient diagnosis was associated with higher risk of disseminated stage (2.21 [1.98, 2.47]) while urgent care was associated with lower risk of disseminated stage (0.22 [0.17, 0.29]). Emergency department diagnosis was associated with higher odds of neurological Lyme disease (1.47 [1.07, 2.02]). Higher risk of disseminated Lyme disease, particularly Lyme arthritis, were observed in cases diagnosed in winter (4.54 [3.84, 5.37]), followed by fall (2.75 [2.35, 3.22]) and spring (2.53 [2.10, 3.04]), compared to summer.

**Table 2:** Adjusted associations (risk ratio, 95% confidence interval) of independent variables with Lyme disease stage (disseminated vs. early stage)

	All disseminated vs. early Lyme disease	Disseminated manifestation vs. early Lyme disease				
		Arthritis	Neurological manifestations	Carditis	Secondary erythema migrans	Other disseminated
	1379/3151 (disseminated vs. early)	762/3151 (arthritis vs. early)	426/3151 (neurological vs. early)	76/2446^a^ (carditis vs. early)	95/3120^a^ (secondary erythema migrans vs. early)	76/3151 (other disseminated vs. early)
Age (years)						
0 to < 10	1.24 (1.08, 1.42)	1.25 (1.05, 1.48)	1.04 (0.77, 1.39)	0.39 (0.13, 1.21)	2.59 (1.47, 4.57)	2.66 (1.33, 5.35)
10 to < 20	1.44 (1.28, 1.63)	1.38 (1.16, 1.63)	1.61 (1.24, 2.10)	1.45 (0.83, 2.55)	2.41 (1.26, 4.58)	2.56 (1.23, 5.34)
20 to < 30	1.21 (1.00, 1.46)	1.16 (0.89, 1.51)	1.41 (0.99, 2.02)	2.01 (0.94, 4.31)	1.54 (0.63, 3.75)	0.31 (0.04, 2.19)
30 to < 50	1.13 (0.99, 1.29)	0.99 (0.82, 1.21)	1.39 (1.09, 1.77)	1.30 (0.75, 2.24)	0.64 (0.30, 1.36)	1.30 (0.64, 2.65)
50 to < 70	1.00 (Reference)	1.00 (Reference)	1.00 (Reference)	1.00 (Reference)	1.00 (Reference)	1.00 (Reference)
70+	1.14 (0.96, 1.35)	1.13 (0.88, 1.47)	1.08 (0.77, 1.53)	1.00 (0.43, 2.36)	1.57 (0.73, 3.38)	1.20 (0.46, 3.08)
Sex						
Male	1.00 (Reference)	1.00 (Reference)	1.00 (Reference)	1.00 (Reference)	1.00 (Reference)	1.00 (Reference)
Female	1.01 (0.93, 1.09)	1.00 (0.89, 1.12)	1.06 (0.90, 1.25)	0.94 (0.55, 1.61)	1.20 (0.83, 1.74)	1.40 (0.88, 2.21)
Race/ethnicity						
White	1.00 (Reference)	1.00 (Reference)	1.00 (Reference)	1.00 (Reference)	1.00 (Reference)	1.00 (Reference)
Non-White	1.25 (0.93, 1.67)	1.18 (0.78, 1.80)	1.33 (0.71, 2.49)	NA	0.93 (0.13, 6.57)	2.07 (0.72, 5.98)
Medical assistance						
0% time	1.00 (Reference)	1.00 (Reference)	1.00 (Reference)	1.00 (Reference)	1.00 (Reference)	1.00 (Reference)
> 0% to < 50% time	0.97 (0.85, 1.11)	0.94 (0.78, 1.14)	0.87 (0.65, 1.16)	1.03 (0.56, 1.89)	0.85 (0.48, 1.49)	1.66 (0.97, 2.84)
≥ 50% time	1.20 (1.05, 1.37)	1.37 (1.15, 1.64)	1.22 (0.93, 1.60)	1.04 (0.47, 2.27)	0.54 (0.24, 1.22)	0.15 (0.02, 1.16)
Primary care contact						
No	1.00 (Reference)	1.00 (Reference)	1.00 (Reference)	1.00 (Reference)	1.00 (Reference)	1.00 (Reference)
Yes	0.59 (0.54, 0.64)	0.55 (0.48, 0.63)	0.44 (0.36, 0.54)	0.51 (0.30, 0.86)	0.75 (0.42, 1.34)	0.13 (0.08, 0.20)
Setting of diagnosis						
Outpatient	1.00 (Reference)	1.00 (Reference)	1.00 (Reference)	1.00 (Reference)	1.00 (Reference)	1.00 (Reference)
Urgent care	0.22 (0.17, 0.29)	0.22 (0.15, 0.31)	0.12 (0.06, 0.21)	NA	0.47 (0.23, 0.93)	0.027 (0.003, 0.21)
Emergency	1.04 (0.85, 1.27)	0.73 (0.50, 1.06)	1.49 (1.09, 2.04)	1.30 (0.48, 3.54)	0.87 (0.31, 2.45)	0.51 (0.16, 1.60)
Inpatient	2.21 (1.98, 2.47)	1.88 (1.49, 2.36)	4.12 (3.30, 5.14)	25.4 (15.22, 42.50)	NA	3.84 (1.59, 9.25)
Season of diagnosis						
Winter (Dec–Feb)	2.19 (1.96, 2.45)	4.54 (3.84, 5.37)	1.45 (1.03, 2.03)	0.78 (0.25, 2.41)	1.17 (0.44, 3.17)	1.25 (0.54, 2.91)
Spring (March–May)	1.35 (1.18, 1.54)	2.53 (2.10, 3.04)	0.77 (0.57, 1.05)	0.70 (0.25, 1.97)	0.69 (0.34, 1.39)	0.34 (0.11, 1.06)
Summer (June–Aug)	1.00 (Reference)	1.00 (Reference)	1.00 (Reference)	1.00 (Reference)	1.00 (Reference)	1.00 (Reference)
Fall (Sept–Nov)	1.46 (1.33, 1.62)	2.75 (2.35, 3.22)	0.84 (0.67, 1.07)	1.31 (0.80, 2.16)	0.75 (0.42, 1.33)	0.92 (0.54, 1.56)
Urban/rural residence						
Rural	1.00 (Reference)	1.00 (Reference)	1.00 (Reference)	1.00 (Reference)	1.00 (Reference)	1.00 (Reference)
Urban cluster	1.10 (0.99, 1.22)	1.13 (0.97, 1.32)	1.20 (0.95, 1.52)	1.29 (0.70, 2.39)	1.33 (0.83, 2.14)	1.18 (0.66, 2.13)
Urbanized area	1.09 (0.98, 1.21)	1.09 (0.94, 1.26)	1.11 (0.90, 1.38)	1.42 (0.95, 2.12)	0.95 (0.54, 1.68)	1.32 (0.77, 2.26)

*NA* not available

^a^No persons with Lyme carditis were non-White and none were diagnosed in the urgent care setting; therefore, non-cases (i.e., early stage cases) who were Non-White or diagnosed in the urgent care setting were excluded from the carditis model. No persons with secondary erythema migrans were diagnosed in the inpatient setting; therefore, early stage cases who were diagnosed in the inpatient setting were excluded from the secondary erythema migrans model

In sensitivity analyses to address possible misclassification of our Lyme disease definition, inferences were similar to the main analysis when we required disseminated cases to have positive serology (IgG or IgM, 45% of disseminated cases) (Additional file 1: Table S5), or an antibiotic order within 30 days before or after the diagnosis date (Additional file 1: Table S6). In exploratory models of effect modification, we found statistical interactions between time on Medical Assistance and setting of diagnosis (p = 0.007) and season and setting of diagnosis (p < 0.001); however, the associations were qualitatively similar to the overall model (results not shown).

## Discussion

In this study, we used clinical data in diagnoses and narrative text from the Geisinger EHR to classify 62% of Lyme disease cases by disease stage and manifestation. Diagnoses, particularly the EDG names that could specifically identify all early and disseminated manifestations, were able to classify 48% of cases. With a novel rule-based text-matching algorithm, we extracted staging information from narrative free text, and classified an additional 13% of cases that could not be classified using diagnoses alone. We observed similar proportions of Lyme disease manifestations compared to the patterns observed in surveillance data and identified novel associations with SES- and health care-related variables. Medical Assistance, a proxy of low SES, was associated with higher odds of disseminated disease, while primary care contact and diagnosis in the urgent care setting (compared to outpatient) were associated with lower odds of disseminated disease. These findings inform future research to determine whether improvements in SES or healthcare access can improve timely diagnosis and treatment of Lyme disease and whether targeted interventions on these factors could prevent disseminated disease.

In this study, we used both diagnoses and narrative free text to identify stage and manifestations of Lyme disease. EDG names could identify all early and disseminated Lyme disease endpoints. With ICD-10, a provider could specify a disseminated Lyme manifestation (e.g., arthritis, meningitis, other neurologic endpoints [A69.2x]), whereas with ICD-9, a provider needed both a generic Lyme disease code (088.81) and a co-diagnosis (e.g., arthritis [711.8x]) to indicate a specific manifestation. Changes in available diagnoses could have influenced classification trends over time; however, EDG names and ICD-10 codes were available throughout the study period, while ICD-9 codes were used for the small proportion of encounters (< 10%) where they were available (inpatient and emergency encounters before 2015). Over the study period, the proportion of all Lyme disease cases staging-relevant information in free text increased from 21 to 55%. By the end of the study, 18% of diagnoses (27% of classified by stage) had staging-relevant information in free text that was not available in diagnoses. We hypothesize that these temporal trends were largely the result of concurrent administrative and legal incentives to increase the volume and richness of clinical information in narrative free text. Historically, most EHR-based epidemiologic studies have identified clinical outcomes using ICD diagnosis codes alone, but extracting information from free text is increasingly common [19]. EHR studies of Lyme disease, however, have previously used narrative text only to identify erythema migrans [25, 26]. Our results suggest that EHR free text can yield valuable information on Lyme disease stage and manifestations beyond what is available from diagnoses alone.

This study classified 70% of Lyme disease cases who could be staged as early stage and 30% as disseminated stage. Importantly, the approximately 90% PPV observed in the validation sample was consistent with a common acceptable level for validation of EHR algorithms [38]. This distribution is in line with national surveillance data, where 72% of confirmed Lyme disease cases had erythema migrans and 28% had at least one disseminated manifestation, with arthritis being the most common (28%), followed by neurologic endpoints (13%) and carditis (2%) [11]. Information on manifestation was only available for 60% of confirmed surveillance cases [11], which is comparable to 62% of cases classified in our study. A recent claims data study from high-incidence US states categorized only 6% of cases as disseminated stage; however, this is likely an underestimate because they identified disseminated cases by a clinically relevant billing code (e.g., arthritis, facial palsy) within 30 days of a generic Lyme disease diagnosis and assumed cases that did not meet these criteria were early stage [10].

We observed similar patterns across age, sex, and season of diagnosis across Lyme disease manifestations as observed in prior studies. In national surveillance data, the frequency of Lyme arthritis is more common among children and adolescents [11] while carditis is more common in young adults, especially young men [16]. Our observations of similar proportions of manifestations to national surveillance for Lyme disease, which is known to over-represent more severe, disseminated cases [24, 39], could suggest common sources of bias less health care provider documentation of uncomplicated, less severe cases in the EHR.

In a Lyme disease vaccine clinical trial, only 2–3% of the 296 definite, possible, or asymptomatic Lyme disease cases developed disseminated manifestations [4]. In a study of 88,022 persons diagnosed with Lyme disease in claims data in high-incidence states, 2.8% cases had arthritis, 2.7% had neurologic manifestations, and < 1% had carditis [10]. Neither study identified secondary erythema migrans. In claims data, the incidence of facial palsy was highest in young men 10–14 years, a newly identified high risk group [10]. We found that most Lyme disease cases were diagnosed in the summer, with the exception of Lyme arthritis, which was more evenly distributed throughout the fall and winter. The strong seasonal association with disseminated Lyme disease, especially the increased odds of Lyme arthritis in winter, is in line with prior surveillance findings that arthritis is the most common manifestation among Lyme disease cases with illness onset during December to March [11].

No prior quantitative epidemiologic studies have examined the relation between SES or health care factors and risk of Lyme disease manifestations. In this study, we observed associations with MA, primary care contact, and setting of diagnosis. Eligibility for MA is determined by federal and state poverty thresholds [40], and is used as an indicator of low SES in EHR studies [31]. Individuals who regularly see a primary care provider may be less likely to delay care. The higher risk of disseminated disease in inpatient settings likely reflects the acute severity of some manifestations, particularly Lyme carditis, which can be fatal, or neurologic symptoms like facial nerve palsy. In prior qualitative research with Geisinger Lyme disease patients, delayed diagnosis and treatment was attributed to appraisal delays (e.g., due to symptom misattribution, intermittent symptoms, atypical or no erythema migrans), behavioral delays in seeking care (e.g., due to inadequate health insurance) and misdiagnosis in urgent care or emergency settings [41]. The protective association observed here between urgent care and disseminated disease suggests that erythema migrans can often be reliably diagnosed in urgent care. We speculate that misdiagnosis in urgent or emergency settings would be more likely with atypical erythema migrans or in cases without any rash, which we could not reliably assess in this study.

This study had some limitations. We could not account for care provided outside of Geisinger, which could be a source of missing data, although the Geisinger health system provides primary, specialty, urgent, and emergency health care services. This could explain our observations of some Lyme disease diagnoses without antibiotic treatment or disseminated stage cases without a record of positive serology, although misclassification of Lyme disease stage is also a possibility. However, sensitivity analyses restricting to persons with antibiotic orders and disseminated diagnoses with positive serology did not affect our inferences. We were not able to classify 38% of Lyme disease cases by stage of disease. The differences in demographic and health care-related variables we observed between Lyme disease cases that could and could not be classified by stage may have influenced our results. However, the percentage of unclassified cases decreased over the study period, as more specific diagnoses were recorded and the richness of narrative free text increased over time. Free text notes were not available from the inpatient setting, which may have resulted in disseminated stage case misclassification, or from phone calls, which may have resulted in early stage case misclassification. While we accounted for common spelling errors, abbreviations, and excluded simple instances of negations or diagnoses not related to the patient in the free text algorithm, extracting accurate clinical information from free text encounter notes is notoriously challenging due to nonstandard grammar, common shorthand and misspelling, and auto-generated text strings [42]. Diagnostic coding accuracy likely varies by provider characteristics, and setting, with higher accuracy for inpatient diagnoses that are often updated at discharge [43]. Using EDG name diagnoses, in addition to ICD-9 and ICD-10 diagnosis codes, allowed for increased flexibility and specificity of Lyme disease manifestations. Although EDG name diagnoses may have limited generalizability to non-Epic EHR, Epic is one of the largest providers of EHR to hospitals in the US. Geisinger’s primary care population is representative of the region’s general population in terms of age and sex [44]; however, findings from a largely rural and suburban, majority non-Hispanic white population may not be transportable to other populations.

## Conclusions

This is the first study to categorize Lyme disease cases by clinical stage and manifestation using both diagnoses and narrative text data from the EHR. Methods for identifying Lyme disease cases by stage and manifestation are critical for Lyme disease epidemiology, for both surveillance and inferential analyses. We found novel evidence that lower SES was associated with higher risk of disseminated Lyme disease, while primary care contact and diagnosis in the urgent care setting were consistently associated with lower risk of disseminated manifestations. Early Lyme disease causes relatively mild symptoms, and uncomplicated cases often respond well to short courses of oral antibiotics. In contrast, disseminated Lyme disease can be severe enough to require hospitalization, and in rare cases of Lyme carditis, can be difficult to treat or even fatal [45]. Delayed diagnosis, which makes disseminated infection more likely, is also a risk factors for post-treatment Lyme disease syndrome [46, 47]. Public health interventions to prevent progression to disseminated stages of Lyme disease, especially in vulnerable groups, are necessary to reduce the substantial health care costs of Lyme disease [48].

## Supplementary Information

**Additional file 1: Table S1.** Lyme disease diagnoses used to identify Lyme disease cases and to classify by clinical stage and manifestation. **Table S2.** Co-diagnoses used to identify Lyme disease cases and to classify by clinical stage and manifestation. **Table S3.** Selected characteristics of 4530 Lyme disease cases classified by stage, by source of staging information. **Table S4.** Selected characteristics of 7310 Lyme disease cases, classified (n = 4530) vs. not classified (n = 2870) by stage. **Table S5.** Sensitivity analysis: adjusted associations (risk ratio, 95% confidence interval) of independent variables with Lyme disease stage (disseminated vs. early stage), excluding disseminated Lyme disease cases without an IgG+ western blot OR EIA+/IgM+ western blot within ± 30 days of Lyme disease diagnosis. **Table S6.** Sensitivity analysis: adjusted associations (risk ratio, 95% confidence interval) of independent variables with Lyme disease stage (disseminated vs. early stage), excluding diagnoses without an appropriate antibiotic order within ± 30 days of Lyme disease diagnosis.

## Data Availability

Individual-level medical record data containing protected health information are available with Geisinger IRB approval and a data use agreement.
