# Supplementary material for: Risk factors for Lyme disease stage and manifestation using electronic health records
Source: BMC Infect Dis. 2021 Dec 20;21:1269. doi: 10.1186/s12879-021-06959-y (PMC8686252; doi:10.1186/s12879-021-06959-y)
Supplement: Supplementary file 1 — Additional file 1: Table S1. Lyme disease diagnoses used to identify Lyme disease cases and to classify by clinical stage and manifestation. Table S2. Co-diagnoses used to identify Lyme disease cases and to classify by clinical stage and manifestation. Table S3. Selected characteristics of 4530 Lyme disease cases classified by stage, by source of staging information. Table S4. Selected characteristics of 7310 Lyme disease cases, classified (n = 4530) vs. not classified (n = 2870) by stage. Table S5. Sensitivity analysis: adjusted associations (risk ratio, 95% confidence interval) of independent variables with Lyme disease stage (disseminated vs. early stage), excluding disseminated Lyme disease cases without an IgG+ western blot OR EIA+/IgM+ western blot within ± 30 days of Lyme disease diagnosis. Table S6. Sensitivity analysis: adjusted associations (risk ratio, 95% confidence interval) of independent variables with Lyme disease stage (disseminated vs. early stage), excluding diagnoses without an appropriate antibiotic order within ± 30 days of Lyme disease diagnosis. [file 12879_2021_6959_MOESM1_ESM.pdf]

Additional File 1

Risk factors for Lyme disease stage and manifestation using electronic health records

Katherine A. Moon[1], Jonathan S. Pollak[1], Melissa N. Poulsen[1, 2], Christopher D. Heaney[1, 2, 3], Annemarie G. Hirsch[1, 2], Brian S. Schwartz[1, 2, 3, 4]

Affiliations:
[1] Department of Environmental Health and Engineering, Johns Hopkins School of Public Health, Baltimore, MD, USA
[2] Department of Population Health Sciences, Geisinger, Danville, PA, USA
[3] Department of Epidemiology, Johns Hopkins School of Public Health, Baltimore, MD, USA
[4] Johns Hopkins School of Medicine, Department of Medicine, Baltimore, MD, USA

Correspondence:
Katherine A. Moon, Dept. of Environmental Health and Engineering, 615 N Wolfe St., W7604, Baltimore, MD USA; kmoon9@jhu.edu

Contents

Table S1. Lyme disease diagnoses used to identify Lyme disease cases and to classify by clinical stage and manifestation 2

Table S2. Co-diagnoses used to identify Lyme disease cases and to classify by clinical stage and manifestation 4

Table S3. Selected characteristics of 4,530 Lyme disease cases classified by stage, by source of staging information 8

Table S4. Selected characteristics of 7,310 Lyme disease cases, classified (n=4530) vs. not classified (n=2870) by stage 9

Table S5. Sensitivity analysis: Adjusted associations (risk ratio, 95% confidence interval) of independent variables with Lyme disease stage (disseminated vs. early stage), *excluding disseminated Lyme disease cases without an IgG+ western blot OR EIA+/IgM+ western blot within +/- 30 days of Lyme disease diagnosis* 10

Table S6. Sensitivity analysis: Adjusted associations (risk ratio, 95% confidence interval) of independent variables with Lyme disease stage (disseminated vs. early stage), *excluding diagnoses without an appropriate antibiotic order within +/- 30 days of Lyme disease diagnosis* 12

Table S1. Lyme disease diagnoses used to identify Lyme disease cases and to classify by clinical stage and manifestation

Diagnoses (dx_nm field)	Source	Diagnosis code
**Lyme disease: Unknown stage**		
LYME DISEASE	ICD-9	088.81
LYME DISEASE, UNSPECIFIED	ICD-10	A69.20
OTHER CONDITIONS ASSOCIATED WITH LYME DISEASE	ICD-10	A69.29
LYME DISEASE	EDG name	1062
LYME BORRELIOSIS	EDG name	51750
JOINT SWELLING DUE TO LYME DISEASE	EDG name	253700
SUSPECTED LYME DISEASE	EDG name	1259993
JOINT PAIN AND SWELLING DUE TO LYME DISEASE	EDG name	379499
LYME DISEASE, UNSPECIFIED	EDG name	1183995
JOINT PAIN DUE TO LYME DISEASE W/LARGEST SKIN LESION 2 INCHES OR MORE	EDG name	375389
JOINT PAIN DUE TO LYME DISEASE	EDG name	253699
JOINT PAIN DUE TO ACUTE LYME DISEASE	EDG name	251779
OTHER NEUROLOGIC DISORDERS IN LYME DISEASE	EDG name	1178339
CONGENITAL LYME DISEASE	EDG name	415117
LYME DISEASE OF INNER EAR	EDG name	414522
**Lyme disease: Early stage**		
EARLY LOCALIZED LYME INFECTION	EDG name	123872
STAGE 1 LYME DISEASE	EDG name	124463
ACUTE LYME DISEASE	EDG name	251903
LYME DISEASE, ACUTE	EDG name	251904
ACUTE LYME DISEASE WITH LARGEST SKIN LESION OF 2 INCHES OR MORE	EDG name	251905
LYME DISEASE WITH LARGEST SKIN LESION LESS THAN 2 INCHES	EDG name	255979
LYME DISEASE WITH LARGEST SKIN LESION OF 2 INCHES OR MORE	EDG name	255980
LYME DERMATITIS	EDG name	426394
EARLY LOCALIZED LYME DISEASE	EDG name	499919
**Lyme disease: Disseminated stage**		
DISSEMINATED LYME DISEASE	EDG name	133898
LATE EFFECT OF LYME DISEASE	EDG name	253782
**Early Lyme disease: Erythema migrans**		
ECM (ERYTHEMA CHRONICUM MIGRANS)	EDG name	107403
SUSPECTED ERYTHEMA CHRONICA MIGRANS	EDG name	1274695
ERYTHEMA MIGRANS (LYME DISEASE)	EDG name	148046
ERYTHEMA CHRONICUM MIGRANS	EDG name	15928
ERYTHEMA CHRONICUM MIGRANS WITH LARGEST SKIN LESION 5 CM OR MORE	EDG name	253133
ERYTHEMA CHRONICUM MIGRANS WITH LARGEST SKIN LESION LESS THAN 5 CM	EDG name	253134
PRIMARY ERYTHEMA CHRONICUM MIGRANS	EDG name	254903

Diagnoses (dx_nm field)	Source	Diagnosis code
PRIMARY ERYTHEMA CHRONICUM MIGRANS W/LARGEST SKIN LESION 5 CM OR MORE	EDG name	374829
ERYTHEMA CHRONICUM MIGRANS WITH LARGEST SKIN LESION 5 CM OR MORE, WITH JOINT PAIN	EDG name	379061
ERYTHEMA CHRONICUM MIGRANS W/JOINT PAIN, LARGEST LESION 5CM OR MORE	EDG name	380100
**Disseminated Lyme disease: Arthritis**		
ARTHRITIS DUE TO LYME DISEASE	ICD-10	A69.23
ARTHRITIS IN LYME DISEASE (HCC)	EDG name	104198
LYME ARTHRITIS OF ELBOW (HCC)	EDG name	200372
LYME ARTHRITIS OF HIP (HCC)	EDG name	200374
LYME ARTHRITIS OF KNEE (HCC)	EDG name	200375
LYME ARTHRITIS OF LOWER LEG (HCC)	EDG name	251077
LYME ARTHRITIS OF WRIST (HCC)	EDG name	251089
LYME ARTHRITIS OF KNEE OR LOWER LEG	EDG name	251807
LYME ARTHRITIS OF LUMBAR SPINE (HCC)	EDG name	255977
LYME ARTHRITIS OF NECK (HCC)	EDG name	255978
ARTHRITIS DUE TO LYME DISEASE (HCC)	EDG name	267003
LYME ARTHRITIS OF FOOT (HCC)	EDG name	324462
LYME ARTHRITIS OF HAND (HCC)	EDG name	324464
LYME ARTHRITIS OF LOW BACK (HCC)	EDG name	324469
LYME ARTHRITIS OF SHOULDER (HCC)	EDG name	324470
LYME ARTHRITIS OF SHOULDER REGION (HCC)	EDG name	324480
LYME ARTHRITIS OF PELVIC REGION AND THIGH (HCC)	EDG name	327274
LYME ARTHRITIS OF MULTIPLE JOINTS (HCC)	EDG name	327277
LYME ARTHRITIS (HCC)	EDG name	36154
LYME ARTHRITIS OF FOREARM (HCC)	EDG name	424406
**Disseminated Lyme disease: Neurological manifestations**		
MENINGITIS DUE TO LYME DISEASE	ICD-10	A69.21
OTHER NEUROLOGIC DISORDERS IN LYME DISEASE	ICD-10	A69.22
NEUROLOGICAL LYME DISEASE	EDG name	157332
ACUTE LYME DISEASE WITH NEUROLOGICAL DISEASE	EDG name	251906
CNS LYME DISEASE	EDG name	325052
LYME MENINGITIS	EDG name	146495
MENINGITIS DUE TO LYME DISEASE	EDG name	146497
LYME NEUROPATHY	EDG name	146821
**Disseminated Lyme disease: Carditis**		
ACUTE PERICARDITIS ASSOCIATED WITH LYME DISEASE	EDG name	1111871
LYME CARDITIS	EDG name	36155
**Disseminated Lyme disease: Other manifestations**		
SECONDARY ERYTHEMA CHRONICUM MIGRANS	EDG name	255125
ERYTHEMA CHRONICA MIGRANS, SECONDARY	EDG name	255126
SECONDARY ERYTHEMA CHRONICUM MIGRANS WITH LARGEST SKIN LESION 5 CM OR MORE	EDG name	343967

Diagnoses (dx_nm field)	Source	Diagnosis code
LYME UVEITIS	EDG name	407276

Table S2. Co-diagnoses used to identify Lyme disease cases and to classify by clinical stage and manifestation

Diagnoses (dx_nm field)	Source	Diagnosis code
**Co-diagnosis codes for Lyme disease manifestations: Rash, dermatitis, or cellulitis**		
RASH AND OTHER NONSPECIFIC SKIN ERUPTION	ICD-9	782.1
CELLULITIS OF LEFT LOWER LIMB	ICD-10	L03.116
CELLULITIS OF RIGHT LOWER LIMB	ICD-10	L03.115
RASH AND OTHER NONSPECIFIC SKIN ERUPTION	ICD-10	R21
CELLULITIS OF ABDOMINAL WALL	ICD-10	L03.311
CELLULITIS OF BUTTOCK	ICD-10	L03.317
CELLULITIS OF BACK (ANY PART EXCEPT BUTTOCK)	ICD-10	L03.312
DERMATITIS	EDG name	7898
RASH AND NONSPECIFIC SKIN ERUPTION	EDG name	9760
CELLULITIS	EDG name	7857
CELLULITIS OF LEG	EDG name	7854
CELLULITIS OF FOOT	EDG name	7855
CELLULITIS OF ARM	EDG name	7851
CELLULITIS OF FACE	EDG name	7848
CELLULITIS OF TRUNK	EDG name	7850
CELLULITIS OF HAND	EDG name	7852
CELLULITIS OF TOE	EDG name	7844
RASH	EDG name	47156
CELLULITIS OF LEFT LOWER EXTREMITY	EDG name	284732
CELLULITIS OF RIGHT LOWER EXTREMITY	EDG name	275130
RASH AND OTHER NONSPECIFIC SKIN ERUPTION	EDG name	55311
CELLULITIS, UNSPECIFIED CELLULITIS SITE	EDG name	1241926
CELLULITIS OF ABDOMINAL WALL	EDG name	19223
CELLULITIS OF LOWER EXTREMITY, UNSPECIFIED LATERALITY	EDG name	1039674
CELLULITIS OF LEFT UPPER EXTREMITY	EDG name	282635
CELLULITIS, LEG	EDG name	105276
CELLULITIS OF RIGHT FOOT	EDG name	243174
CELLULITIS OF RIGHT LOWER LEG	EDG name	365189
RASH, SKIN	EDG name	101769
CELLULITIS OF ARM, RIGHT	EDG name	243166
CELLULITIS OF ARM, LEFT	EDG name	243129
CELLULITIS, FACE	EDG name	105274
CELLULITIS OF LEFT HAND	EDG name	243145
CELLULITIS OF RIGHT HAND	EDG name	243182

Diagnoses (dx_nm field)	Source	Diagnosis code
CELLULITIS OF RIGHT THIGH	EDG name	365192
CELLULITIS OF GROIN	EDG name	19227
RASH/SKIN ERUPTION	EDG name	220704
CELLULITIS OF HEAD EXCEPT FACE	EDG name	250266
CELLULITIS OF BACK	EDG name	235774
CELLULITIS OF TRUNK, UNSPECIFIED SITE OF TRUNK	EDG name	1241097
RASH OF ENTIRE BODY	EDG name	418725
CELLULITIS OF SHOULDER	EDG name	19234
CELLULITIS OF FOREARM, RIGHT	EDG name	243179
RASH OF BACK	EDG name	405409
TARGET RASH	EDG name	227121
CELLULITIS OF AXILLA, RIGHT	EDG name	243168
MACULAR ERYTHEMATOUS RASH	EDG name	326426
LOCALIZED RASH	EDG name	263501
CELLULITIS OF LEFT EAR	EDG name	466784
CELLULITIS OF CHEEK	EDG name	252463
CELLULITIS OF AXILLA	EDG name	19233
ATYPICAL RASH	EDG name	256681
CELLULITIS OF INDEX FINGER, LEFT	EDG name	764254
**Co-diagnosis codes for Lyme disease manifestations: Arthritis**		
ARTHROPATHY ASSOCIATED WITH OTHER INFECTIOUS AND PARASITIC DISEASES, LOWER LEG	ICD-9	711.86
ARTHROPATHY ASSOCIATED WITH OTHER INFECTIOUS AND PARASITIC DISEASES, SITE UNSPECIFIED	ICD-9	711.8
MONOARTHRITIS, NOT ELSEWHERE CLASSIFIED, LEFT KNEE	ICD-10	M13.162
OTHER SPECIFIED ARTHRITIS, UNSPECIFIED ANKLE AND FOOT	ICD-10	M13.879
MONOARTHRITIS, NOT ELSEWHERE CLASSIFIED, UNSPECIFIED SITE	ICD-10	M13.10
ARTHRITIS OF KNEE	EDG name	15319
ARTHRITIS	EDG name	43271
ARTHROPATHY	EDG name	8356
ARTHRITIS OF HIP	EDG name	15318
LOCALIZED, PRIMARY OSTEOARTHRITIS OF ANKLE OR FOOT	EDG name	8232
ARTHROPATHY OF LOWER LEG	EDG name	8362
OSTEOARTHRITIS OF RIGHT KNEE, UNSPECIFIED OSTEOARTHRITIS TYPE	EDG name	1039714
OSTEOARTHRITIS OF LEFT KNEE, UNSPECIFIED OSTEOARTHRITIS TYPE	EDG name	1039544
INFLAMMATORY ARTHRITIS	EDG name	205668
ARTHRITIS OF KNEE, RIGHT	EDG name	238648
ARTHRITIS OF KNEE, LEFT	EDG name	238634
UNSPECIFIED MONOARTHRITIS, LOWER LEG	EDG name	8341
KNEE ARTHROPATHY	EDG name	316950
REACTIVE ARTHRITIS (HCC)	EDG name	313161
ARTHRITIS OF LEFT KNEE	EDG name	238633
MONOARTHRITIS OF ANKLE	EDG name	8342

Diagnoses (dx_nm field)	Source	Diagnosis code
INFECTIVE ARTHRITIS (HCC)	EDG name	8129
ARTHROPATHY ASSOCIATED WITH OTHER BACTERIAL DISEASES, LOWER LEG	EDG name	8080
ACUTE ARTHRITIS	EDG name	20124
UNSPECIFIED MONOARTHRITIS, OTHER SPECIFIED SITES	EDG name	8343
OLIGOARTHRITIS	EDG name	207841
ARTHRITIS OF RIGHT KNEE DUE TO OTHER BACTERIA (HCC)	EDG name	1178148
INFECTIVE ARTHRITIS, ANKLE AND FOOT (HCC)	EDG name	8136
INFLAMMATORY MONOARTHRITIS OF KNEE OR LOWER LEG	EDG name	257475
ARTHRITIS, REACTIVE (HCC)	EDG name	313165
**Co-diagnosis codes for Lyme disease manifestations: Neurological**		
BELL'S PALSY	ICD-9	351
MENINGITIS IN OTHER BACTERIAL DISEASES CLASSIFIED ELSEWHERE	ICD-9	320.7
BELL'S PALSY	ICD-10	G51.0
MENINGITIS, UNSPECIFIED	ICD-10	G03.9
BELL'S PALSY	EDG name	3622
MENINGITIS	EDG name	3399
FACIAL NERVE PALSY	EDG name	47312
BELL PALSY	EDG name	47306
FACIAL PALSY	EDG name	31011
LEFT-SIDED BELL'S PALSY	EDG name	506784
RIGHT-SIDED BELL'S PALSY	EDG name	506785
**Co-diagnosis codes for Lyme disease manifestations: Carditis**		
FIRST DEGREE ATRIOVENTRICULAR BLOCK	ICD-9	426.11
ATRIOVENTRICULAR BLOCK, COMPLETE	ICD-9	426
OTHER SECOND-DEGREE ATRIOVENTRICULAR BLOCK	ICD-9	426.13
MOBITZ (TYPE) II ATRIOVENTRICULAR BLOCK	ICD-9	426.12
OTHER HEART BLOCK	ICD-9	426.6
ACUTE PERICARDITIS IN DISEASES CLASSIFIED ELSEWHERE	ICD-9	420
ACUTE MYOCARDITIS IN DISEASES CLASSIFIED ELSEWHERE	ICD-9	422
ATRIOVENTRICULAR BLOCK, FIRST DEGREE	ICD-10	I44.0
ATRIOVENTRICULAR BLOCK, COMPLETE	ICD-10	I44.2
ATRIOVENTRICULAR BLOCK, SECOND DEGREE	ICD-10	I44.1
UNSPECIFIED ATRIOVENTRICULAR BLOCK	ICD-10	I44.30
MYOCARDITIS, UNSPECIFIED	ICD-10	I51.4
ATRIOVENTRICULAR BLOCK, COMPLETE (HCC)	EDG name	5064
COMPLETE HEART BLOCK (HCC)	EDG name	17904
ACUTE PERICARDITIS	EDG name	5023
CHB (COMPLETE HEART BLOCK) (HCC)	EDG name	97074
OTHER HEART BLOCK	EDG name	5079
SECOND DEGREE MOBITZ II AV BLOCK	EDG name	15741
SECOND DEGREE MOBITZ I AV BLOCK	EDG name	15742

Diagnoses (dx_nm field)	Source	Diagnosis code
MYOCARDITIS (HCC)	EDG name	5109
FIRST DEGREE HEART BLOCK	EDG name	17905
FIRST DEGREE AV BLOCK	EDG name	59159
AVB (ATRIOVENTRICULAR BLOCK)	EDG name	96534
HEART BLOCK	EDG name	48934
MOBITZ TYPE I INCOMPLETE ATRIOVENTRICULAR BLOCK	EDG name	17906
MOBITZ TYPE II ATRIOVENTRICULAR BLOCK	EDG name	17908
HEART BLOCK AV SECOND DEGREE	EDG name	109135
ACUTE MYOCARDITIS	EDG name	5033
AV BLOCK, 1ST DEGREE	EDG name	84727
HEART BLOCK AV COMPLETE (HCC)	EDG name	99053
ATRIOVENTRICULAR BLOCK	EDG name	5066
AV BLOCK	EDG name	43448
MOBITZ TYPE 1 SECOND DEGREE ATRIOVENTRICULAR BLOCK	EDG name	111542
AV HEART BLOCK	EDG name	184111
TRANSIENT COMPLETE HEART BLOCK (HCC)	EDG name	206859
INTERMITTENT COMPLETE HEART BLOCK (HCC)	EDG name	379203
AV BLOCK, MOBITZ 1	EDG name	234898
PERICARDITIS, ACUTE	EDG name	62012
FIRST DEGREE HEART BLOCK BY ELECTROCARDIOGRAM	EDG name	433709
AV BLOCK, MOBITZ II	EDG name	84728

Table S3. Selected characteristics of 4,530 Lyme disease cases classified by stage, by source of staging information

Characteristic	Classified by diagnosis & free text n = 1,671 (36.9%)	Classified by diagnosis only n=1,902 (42.0%)	Classified by free text only n=957 (21.1%)	p-value (a)
Disseminated stage, n (%)				
No	1171 (37.2%)	1324 (42.0%)	656 (20.8%)	0.71
Yes	500 (36.3%)	578 (41.9%)	301 (21.8%)	
Year of diagnosis, n (%)				
2012	118 (21.2%)	334 (60.1%)	104 (18.7%)	< 0.001
2013	131 (17.2%)	515 (67.6%)	116 (15.2%)	
2014	207 (23.4%)	542 (61.2%)	136 (15.4%)	
2015	539 (51.7%)	245 (23.5%)	259 (24.8%)	
2016	676 (52.7%)	266 (20.7%)	342 (26.6%)	
Age, years, median (IQR)	35.9 (10.4, 58.3)	38.9 (12.1, 59.5)	41.1 (14.2, 59.9)	0.002
Age, n (%)				
< 10 years	394 (42.0%)	376 (40.1%)	168 (17.9%)	0.008
10 to < 20 years	244 (37.8%)	272 (42.1%)	129 (20.0%)	
20 to < 30 years	111 (32.3%)	150 (43.6%)	83 (24.1%)	
30 to < 50 years	318 (35.7%)	387 (43.4%)	186 (20.9%)	
50 to < 70 years	469 (36.5%)	528 (41.1%)	288 (22.4%)	
> 70 years	135 (31.6%)	189 (44.3%)	103 (24.1%)	
Sex				
Male, n (%)	923 (37.1%)	1043 (42.0%)	520 (20.9%)	0.90
Female, n (%)	748 (36.6%)	859 (42.0%)	437 (21.4%)	
Race/ethnicity, n (%)				
White	1648 (36.9%)	1872 (41.9%)	951 (21.3%)	0.10
Non-White	23 (39.0%)	30 (50.9%)	6 (10.2%)	
Season of diagnosis, n (%)				
Winter	96 (33.1%)	123 (42.4%)	71 (24.5%)	0.45
Spring	186 (34.6%)	242 (45.1%)	109 (20.3%)	
Summer	1044 (37.8%)	1142 (41.3%)	577 (20.9%)	
Fall	345 (36.7%)	395 (42.0%)	200 (21.3%)	
Setting of diagnosis, n (%)				
Outpatient	1163 (34.0%)	1457 (42.6%)	802 (23.4%)	<0.001
Urgent care	408 (55.7%)	251 (34.2%)	74 (10.1%)	
Emergency	59 (28.4%)	77 (37.0%)	72 (34.6%)	
Inpatient	41 (24.6%)	117 (70.1%)	9 (5.4%)	
Primary care contact, n (%)				
No	361 (37.5%)	337 (35.0%)	265 (27.5%)	< 0.001
Yes	1310 (36.7%)	1565 (43.9%)	692 (19.4%)	
Medical Assistance, n (%)				
0% time	1340 (80.2%)	1492 (78.4%)	783 (81.8%)	0.024
>0% to <50% time	201 (12.0%)	217 (11.4%)	108 (11.3%)	
≥50% time	130 (7.8%)	193 (10.1%)	66 (6.9%)	
Community type, n (%)				
Township	1271 (37.1%)	1410 (41.2%)	745 (21.8%)	0.061
Borough	325 (35.3%)	411 (44.6%)	186 (20.2%)	
City	75 (41.2%)	81 (44.5%)	26 (14.3%)	
Urban/rural residence, n (%)				
Rural	1031 (36.7%)	1181 (42.1%)	595 (21.2%)	0.28
Urban cluster	261 (37.1%)	312 (44.3%)	131 (18.6%)	
Urbanized area	379 (37.2%)	409 (40.1%)	231 22.7%)	

Notes: IQR, Interquartile range; NA, Not available.
(a) Chi-square p-values for categorical variables. Kruskal-Wallis non-parametric test for continuous variables.

Table S4. Selected characteristics of 7,310 Lyme disease cases, classified (n=4530) vs. not classified (n=2870) by stage

Characteristic	Not classified by stage n = 2,780 (38.0%)	Classified by stage n = 4,530 (62.0%)	p-value (a)
Year of diagnosis, n (%)			
2012	484 (46.5%)	556 (53.5%)	< 0.001
2013	622 (44.9%)	762 (55.1%)	
2014	628 (41.5%)	885 (58.5%)	
2015	464 (30.8%)	1043 (69.2%)	
2016	582 (31.2%)	1284 (68.8%)	
Age, years, median (IQR)	46.1 (24.0, 61.2)	38.3 (11.8, 59.1)	< 0.001
Age, n (%)			
< 10 years	318 (25.3%)	938 (74.7%)	< 0.001
10 to < 20 years	286 (30.7%)	645 (69.3%)	
20 to < 30 years	249 (42.0%)	344 (58.0%)	
30 to < 50 years	701 (44.0%)	891 (56.0%)	
50 to < 70 years	896 (41.1%)	1285 (58.9%)	
> 70 years	330 (43.6%)	427 (56.4%)	
Sex			
Male, n (%)	1482 (37.4%)	2486 (62.7%)	0.19
Female, n (%)	1298 (38.8%)	2044 (61.2%)	
Race/ethnicity, n (%)			
White	2729 (37.9%)	4471 (62.1%)	0.07
Non-White	51 (46.4%)	59 (53.6%)	
Season of diagnosis, n (%)			
Winter	303 (51.1%)	290 (48.9%)	< 0.001
Spring	421 (44.0%)	537 (56.1%)	
Summer	1368 (33.1%)	2763 (66.9%)	
Fall	688 (42.3%)	940 (57.7%)	
Setting of diagnosis, n (%)			
Outpatient	2364 (40.9%)	3422 (59.1%)	< 0.001
Urgent care	114 (13.5%)	733 (86.5%)	
Emergency	191 (47.9%)	208 (52.1%)	
Inpatient	111 (39.9%)	167 (60.1%)	
Primary care contact, n (%)			
No	707 (42.3%)	963 (57.7%)	< 0.001
Yes	2073 (36.8%)	3567 (63.2%)	
Medical Assistance, n (%)			
0% time	2242 (80.6%)	3615 (79.8%)	0.39
>0% to <50% time	294 (10.6%)	526 (11.6%)	
≥50% time	244 (8.8%)	389 (8.6%)	
Community type, n (%)			
Township	2058 (37.5%)	3426 (62.5 %)	0.11
Borough	584 (38.8%)	922 (61.2%)	
City	128 (43.1%)	182 (56.9%)	
Urban/rural residence, n (%)			
Rural	1633 (36.8%)	2807 (63.2%)	0.023
Urban cluster	467 (39.9%)	704 (60.1%)	
Urbanized area	680 (40.0%)	1019 (60.0%)	

Notes: IQR, Interquartile range

(a) Chi-square p-values for categorical variables. Kruskal-Wallis non-parametric test for continuous variables.

Table S5. Sensitivity analysis: Adjusted associations (risk ratio, 95% confidence interval) of independent variables with Lyme disease stage (disseminated vs. early stage), *excluding disseminated Lyme disease cases without an IgG+ western blot <u>OR</u> EIA+/IgM+ western blot within +/- 30 days of Lyme disease diagnosis*

	**Disseminated with positive serology vs. Early** 621 disseminated / 3151 early	**Arthritis with positive serology vs. Early** 333 disseminated / 3151 early	**Neurological manifestations with positive serology vs. Early** 202 disseminated / 3151 early	**Carditis with positive serology vs. Early** 49 disseminated / 2355 early (a)	**Secondary erythema migrans with positive serology vs. Early** 30 disseminated / 2959 early (a)	**"Other disseminated" with positive serology vs. Early** 29 disseminated / 2128 early (a)
Age, years						
0 to < 10 years	1.53 (1.24,1.89)	2.03 (1.53,2.70)	1.10 (0.76,1.60)	0.79 (0.19,3.26)	1.32 (0.42,4.17)	1.78 (0.61,5.23)
10 to < 20 years	1.90 (1.56,2.32)	2.19 (1.64,2.92)	1.64 (1.12,2.40)	2.79 (1.29,6.05)	2.91 (1.05,8.08)	2.00 (0.64,6.21)
20 to < 30 years	1.12 (0.81,1.54)	1.17 (0.72,1.91)	1.09 (0.61,1.94)	2.10 (0.76,5.80)	1.31 (0.25,6.96)	NA
30 to < 50 years	1.00 (0.80,1.25)	0.57 (0.36,0.92)	1.42 (1.00,2.01)	1.60 (0.79,3.23)	0.22 (0.03,1.72)	0.84 (0.28,2.50)
50 to < 70 years	1.00 (Reference)	1.00 (Reference)	1.00 (Reference)	1.00 (Reference)	1.00 (Reference)	1.00 (Reference)
70+ years	1.08 (0.80,1.44)	1.10 (0.69,1.74)	1.04 (0.62,1.72)	0.67 (0.19,2.30)	2.60 (0.96,7.01)	0.57 (0.12,2.61)
Sex						
Male	1.00 (Reference)	1.00 (Reference)	1.00 (Reference)	1.00 (Reference)	1.00 (Reference)	1.00 (Reference)
Female	0.97 (0.86,1.10)	0.91 (0.76,1.09)	1.16 (0.92,1.47)	0.91 (0.46,1.79)	1.37 (0.69,2.70)	2.64 (1.15,6.05)
Race/ethnicity						
White	1.00 (Reference)	1.00 (Reference)	1.00 (Reference)	1.00 (Reference)	1.00 (Reference)	1.00 (Reference)
Non-White	1.56 (1.00,2.45)	1.54 (0.86,2.76)	2.07 (0.86,4.95)	NA	NA	1.63 (0.15,17.60)
Medical Assistance						
0% time	1.00 (Reference)	1.00 (Reference)	1.00 (Reference)	1.00 (Reference)	1.00 (Reference)	1.00 (Reference)
>0% to <50% time	0.87 (0.69,1.08)	0.89 (0.66,1.19)	0.58 (0.36,0.94)	1.11 (0.57,2.17)	0.99 (0.32,3.07)	1.74 (0.64,4.78)
≥50% time	1.38 (1.12,1.70)	1.47 (1.13,1.90)	1.30 (0.88,1.93)	1.32 (0.57,3.04)	0.72 (0.16,3.22)	NA
Primary care contact						
No	1.00 (Reference)	1.00 (Reference)	1.00 (Reference)	1.00 (Reference)	1.00 (Reference)	1.00 (Reference)
Yes	0.69 (0.59,0.82)	0.72 (0.56,0.92)	0.59 (0.43,0.81)	0.81 (0.46,1.43)	1.18 (0.30,4.54)	0.15 (0.07,0.32)
Setting of diagnosis						
Outpatient	1.00 (Reference)	1.00 (Reference)	1.00 (Reference)	1.00 (Reference)	1.00 (Reference)	1.00 (Reference)

	**Disseminated with positive serology vs. Early** 621 disseminated / 3151 early	**Arthritis with positive serology vs. Early** 333 disseminated / 3151 early	**Neurological manifestations with positive serology vs. Early** 202 disseminated / 3151 early	**Carditis with positive serology vs. Early** 49 disseminated / 2355 early (a)	**Secondary erythema migrans with positive serology vs. Early** 30 disseminated / 2959 early (a)	**"Other disseminated" with positive serology vs. Early** 29 disseminated / 2128 early (a)
Urgent care	0.23 (0.16,0.34)	0.24 (0.14,0.41)	0.18 (0.09,0.38)	NA	0.61 (0.20,1.88)	NA
Emergency	1.46 (1.08,1.98)	1.11 (0.71,1.73)	2.55 (1.58,4.12)	3.42 (0.95,12.28)	NA	1.14 (0.30,4.28)
Inpatient	4.34 (3.67,5.13)	2.69 (1.95,3.71)	9.51 (6.95,13.00)	88.4 (42.16,185.49)	NA	9.46 (2.90,30.85)
Season of diagnosis						
Winter	2.57 (2.10,3.15)	6.67 (5.01,8.89)	0.89 (0.47,1.68)	NA	0.83 (0.11,6.37)	0.70 (0.10,5.04)
Spring	1.23 (0.97,1.56)	2.90 (2.12,3.96)	0.26 (0.12,0.56)	0.41 (0.05,3.13)	0.49 (0.11,2.11)	0.47 (0.12,1.94)
Summer	1.00 (Reference)	1.00 (Reference)	1.00 (Reference)	1.00 (Reference)	1.00 (Reference)	1.00 (Reference)
Fall	1.58 (1.35,1.86)	3.81 (3.00,4.86)	0.71 (0.50,1.02)	1.37 (0.74,2.54)	0.63 (0.22,1.84)	0.29 (0.07,1.19)
Urban/rural residence						
Rural	1.00 (Reference)	1.00 (Reference)	1.00 (Reference)	1.00 (Reference)	1.00 (Reference)	1.00 (Reference)
Urban cluster	1.11 (0.90,1.37)	1.14 (0.85,1.54)	1.24 (0.85,1.82)	0.66 (0.37,1.19)	1.58 (0.63,3.95)	1.24 (0.43,3.52)
Urbanized area	1.15 (0.96,1.39)	1.28 (1.01,1.63)	1.00 (0.71,1.39)	1.04 (0.70,1.55)	0.84 (0.32,2.24)	1.05 (0.42,2.60)

Notes: NA, Not available.

(a) For carditis, secondary erythema migrans, and other disseminated manifestations models, some independent variables had missing values and these categories were excluded.

Table S6. Sensitivity analysis: Adjusted associations (risk ratio, 95% confidence interval) of independent variables with Lyme disease stage (disseminated vs. early stage), *excluding diagnoses without an appropriate antibiotic order within +/- 30 days of Lyme disease diagnosis*

	**Disseminated vs. Early** 1135 disseminated / 2977 early	**Arthritis vs. Early** 618 disseminated / 2977 early	**Neurological manifestations vs. Early** 349 disseminated / 2977 early	**Carditis vs. Early** 64 disseminated / 2283 early (a)	**Secondary erythema migrans vs. Early** 88 disseminated / 2950 early (a)	**"Other disseminated" vs. Early** 59 disseminated / 2764 early (a)
Age, years						
0 to < 10 years	1.34 (1.15,1.56)	1.39 (1.15,1.68)	1.05 (0.76,1.47)	0.53 (0.17,1.65)	2.49 (1.40,4.43)	3.99 (1.61,9.88)
10 to < 20 years	1.60 (1.39,1.84)	1.59 (1.31,1.94)	1.73 (1.28,2.33)	1.58 (0.86,2.91)	2.45 (1.28,4.70)	4.63 (1.88,11.39)
20 to < 30 years	1.22 (0.99,1.51)	1.14 (0.84,1.54)	1.45 (1.00,2.10)	1.57 (0.66,3.73)	1.71 (0.70,4.19)	0.52 (0.07,3.98)
30 to < 50 years	1.11 (0.95,1.29)	0.93 (0.72,1.19)	1.39 (1.06,1.81)	1.31 (0.72,2.38)	0.60 (0.28,1.31)	1.66 (0.66,4.20)
50 to < 70 years	1.00 (Reference)	1.00 (Reference)	1.00 (Reference)	1.00 (Reference)	1.00 (Reference)	1.00 (Reference)
70+ years	1.19 (0.99,1.45)	1.29 (0.96,1.73)	1.03 (0.70,1.53)	0.88 (0.34,2.29)	1.55 (0.69,3.50)	2.05 (0.71,5.95)
Sex						
Male	1.00 (Reference)	1.00 (Reference)	1.00 (Reference)	1.00 (Reference)	1.00 (Reference)	1.00 (Reference)
Female	0.99 (0.91,1.09)	0.96 (0.84,1.09)	1.09 (0.90,1.30)	1.10 (0.60,2.03)	1.16 (0.79,1.71)	1.25 (0.72,2.17)
Race/ethnicity						
White	1.00 (Reference)	1.00 (Reference)	1.00 (Reference)	1.00 (Reference)	1.00 (Reference)	1.00 (Reference)
Non-White	1.45 (1.08,1.95)	1.45 (1.00,2.11)	1.67 (0.83,3.34)	NA	1.01 (0.14,7.29)	2.89 (1.12,7.46)
Medical Assistance						
0% time	1.00 (Reference)	1.00 (Reference)	1.00 (Reference)	1.00 (Reference)	1.00 (Reference)	1.00 (Reference)
>0% to <50% time	0.98 (0.85,1.14)	0.98 (0.80,1.20)	0.82 (0.59,1.13)	1.16 (0.62,2.17)	0.83 (0.46,1.49)	1.67 (0.90,3.10)
≥50% time	1.20 (1.04,1.39)	1.35 (1.12,1.64)	1.22 (0.89,1.66)	1.34 (0.60,2.98)	0.58 (0.25,1.32)	NA
Primary care contact						
No	1.00 (Reference)	1.00 (Reference)	1.00 (Reference)	1.00 (Reference)	1.00 (Reference)	1.00 (Reference)
Yes	0.60 (0.54,0.66)	0.53 (0.45,0.61)	0.50 (0.39,0.63)	0.55 (0.31,1.00)	0.87 (0.43,1.74)	0.12 (0.07,0.23)
Setting of diagnosis						
Outpatient	1.00 (Reference)	1.00 (Reference)	1.00 (Reference)	1.00 (Reference)	1.00 (Reference)	1.00 (Reference)

	**Disseminated vs. Early** 1135 disseminated / 2977 early	**Arthritis vs. Early** 618 disseminated / 2977 early	**Neurological manifestations vs. Early** 349 disseminated / 2977 early	**Carditis vs. Early** 64 disseminated / 2283 early (a)	**Secondary erythema migrans vs. Early** 88 disseminated / 2950 early (a)	**"Other disseminated" vs. Early** 59 disseminated / 2764 early (a)
Urgent care	0.24 (0.18,0.32)	0.22 (0.15,0.31)	0.13 (0.07,0.25)	NA	0.50 (0.24,1.03)	0.030 (0.00,0.25)
Emergency	1.09 (0.87,1.37)	0.71 (0.47,1.09)	1.78 (1.28,2.48)	1.97 (0.68,5.75)	0.68 (0.20,2.32)	0.61 (0.18,2.05)
Inpatient	2.49 (2.21,2.81)	2.03 (1.58,2.62)	5.21 (4.08,6.66)	45.2 (25.33,80.59)	NA	4.56 (1.61,12.96)
Season of diagnosis						
Winter	2.29 (2.02,2.60)	4.86 (4.06,5.81)	1.46 (0.97,2.18)	0.55 (0.17,1.78)	1.41 (0.53,3.75)	0.84 (0.25,2.79)
Spring	1.33 (1.15,1.54)	2.46 (1.99,3.04)	0.75 (0.53,1.06)	0.96 (0.28,3.28)	0.63 (0.30,1.35)	0.41 (0.13,1.27)
Summer	1.00 (Reference)	1.00 (Reference)	1.00 (Reference)	1.00 (Reference)	1.00 (Reference)	1.00 (Reference)
Fall	1.50 (1.34,1.68)	2.96 (2.49,3.52)	0.81 (0.61,1.06)	1.79 (1.07,3.02)	0.73 (0.41,1.31)	0.60 (0.29,1.25)
Urban/rural residence						
Rural	1.00 (Reference)	1.00 (Reference)	1.00 (Reference)	1.00 (Reference)	1.00 (Reference)	1.00 (Reference)
Urban cluster	1.09 (0.96,1.23)	1.07 (0.88,1.30)	1.29 (1.00,1.66)	1.07 (0.56,2.03)	1.35 (0.83,2.20)	0.99 (0.47,2.07)
Urbanized area	1.10 (0.98,1.23)	1.16 (1.00,1.34)	1.06 (0.84,1.34)	1.51 (0.99,2.32)	0.90 (0.49,1.65)	1.02 (0.54,1.94)

(a) For carditis, secondary erythema migrans, and other disseminated manifestations models, some independent variables had missing values and these categories were excluded. Excluded variables marked as "NA" in above table.
